# Analyzing mechanisms of interdisciplinary cooperation in promoting students’ health at university

**DOI:** 10.1186/s12889-023-16786-2

**Published:** 2023-10-03

**Authors:** Philip Bachert, Laura Wolbring, Claudia Hildebrand, Alexander Woll, Hagen Wäsche

**Affiliations:** Institute of Sports and Sports Science, Karlsruhe Institute of Technology, Karlsruhe, Germany

**Keywords:** Health-promoting universities, Healthy campus, Network analysis, Ergm, Cooperation

## Abstract

**Background:**

Interdisciplinary cooperation among university actors and resulting intersectoral synergies are considered cornerstones in the process of incorporating health promotion practices in everyday university life in order to break down barriers and provide better access to health promotion services. To date, no network of a health-promoting university has been examined regarding the processes underlying tie formation, network emergence, and maintenance.

**Objectives and methods:**

The goals of this study are to obtain insight into the mechanisms of cooperation between university actors in a health-promoting network and to identify the structural and attributive factors associated with establishing cooperation between actors in the observed network in order to better understand how to build and develop successful networks in the future. For this purpose, a social network analysis was carried out and exponential random graph models were estimated to test corresponding hypotheses.

**Results:**

The network at hand consists of 33 actors (e.g. University Sports Center, General Student Committee) and shows a flat, non-hierarchical structure. Data reveal that attributed competence predicts cooperation (0.32; p < 0.05). Significant homophily effects among student actors (1.31; p < 0.05) and among university actors (0.59; p < 0.05) were found. All structural predictors examined were significant (0.22–5.40; p < 0.05) and are therefore essential in determining the likelihood of cooperation between actors involved in the network.

**Conclusion:**

The results of this study provide for a better understanding of the mechanisms of cooperation and can be used to further develop the network at hand (e.g. selection of key actors for information dissemination or integration of peripheral actors). In addition, the findings offer starting points for sustained network development at other universities (e.g. significance of network governance form or goal consensus). Knowing the factors that influence the network structure, here the conditions of cooperation, results in opportunities to encourage empowerment among actors. However, the analysis of the network undertaken does not directly bear on the success of the network.

**Supplementary Information:**

The online version contains supplementary material available at 10.1186/s12889-023-16786-2.

## Introduction

### Problem and relevance

University students are of particular relevance from a public health perspective [1]. Not only because they represent a considerable proportion of the population in need of health promotion, but especially because of their potential role in promoting health as future leaders, decision makers, and parents [2]. The transition from secondary to tertiary education is a decisive moment causing substantial changes in life and occurs parallel to the transition from adolescence to adulthood [3]. During this critical period of young adulthood, the behavioral habits in the years to come are formed [4].

University students face various stressors, including general academics stressors and exams, lack of time, financial worries, uncertainty of plans after graduation, expectations both of themselves and others, relationship problems, and loneliness [5]. Despite their young age, different health problems, such as stress [6], burnout [7], depression [8], overweight and obesity [9, 10], back pain [11, 12], sleep disorders [13–15], and migraine [16] are common among university students. Moreover, university students tend to engage in risky health behaviors, for example alcohol consumption [17–19], unhealthy eating behavior [20], physical inactivity [21, 22], sedentariness [23], smoking [24, 25], use of other substances [26, 27], internet addiction [28], suicidal thoughts and behaviors [29], and inability to find, understand, evaluate, and apply appropriate health information to make health-related decisions [30]. COVID-19 may have exacerbated existing health issues, for example sleep problems [31] or physical inactivity [32], and the impact of stressors evident prior to the outbreak [33].

The literature available on health-promoting universities shows a wide range of approaches to promoting modifiable health-influencing factors of students [34]. Overall, interventions aiming at the individual level, as opposed to environment-level interventions, are overrepresented [34], likely because implementation and evaluation of environment-level interventions are more complicated [35]. Nevertheless, state-of-the-art models for the explanation of health recognize that health goes beyond the individual level and is affected by environmental characteristics, for example at the organizational level (see socio-ecological frameworks [36, 37]. These findings call for action as regards innovative setting-based strategies to promote health of university students. They confirm numerous opinions underlining the need for a whole-university approach that pays attention to the complex interactions and interconnections between component parts and highlights how the organization can function effectively as a social system [38, 39].

Interdisciplinary cooperation among university actors and resulting intersectoral synergies are considered cornerstones in incorporating health promotion practices into everyday university life in order to break down barriers and provide better access to health promotion services [2, 40–44]. Collective action by a wide range of stakeholders is essential for effective health promotion, since a single stakeholder can hardly be in control of the complex interplay of multifaceted determinants of a targeted population’s health [45–47]. In their study of success factors for a health-promoting university [48], the authors conclude that a well-connected group consisting of various key players working towards health promotion at university would be beneficial. The *Okanagan Charter: An International Charter for Health Promoting Universities and Colleges* developed to promote health within university settings recommends working according to the *setting approach*. This means that relevant stakeholders from various disciplines and sectors within the campus community should be cooperatively involved in the process of embedding health into all aspects of campus culture (e.g. curricula, teaching, research) and of providing health-promoting activities for students. Actors and organizations that are only indirectly concerned with students’ health should also be included.

Partnerships offer multiple benefits, including information exchange, knowledge gain, building trust and increasing reach with the target population, access to and provision of additional resources, avoidance of duplicate structures, boost to innovation, possibility of achieving higher goals, opportunity of task sharing, and pursuit of a holistic approach [45, 49]. Since universities are complex organizations, systematic navigation of health promotion is necessary for them to be effective and efficient [50]. In contrast to traditional social science methods, social network analysis is uniquely suited to this purpose, as it visualizes and describes relationships between actors as well as the overall network structure [51–53].

### State of research

In the past, various intra- and interorganizational public health networks were examined using social network analysis to visualize structural characteristics and cooperation processes, such as active living [54–56], cancer support [57, 58], children’s health initiatives [59, 60], community care [61–63], elderly care [64–66], HIV/AIDS service [67], injury prevention and control [68], mental health services [69–72], physical activity promotion [73–75], prevention of diabetes [76], tobacco control [77–80], and women’s health [81, 82]. These public health networks differ in many ways from the network at hand, because they address the specific health needs and problems of other populations, have a different health-related focus and mission, geographic coverage, types of stakeholders, ways in which network members cooperate, availability and distribution of resources and funding, and political, cultural, and social context.

A multi-methodical, but not network-analytical approach to mapping and characterizing health-promoting structures of an university was used by Sarmiento [41]. Information on localization, resources, and partnerships of health promotion initiatives was collected via semi-structured interviews with stakeholders in health-related roles. As common in literature on health-promoting universities, however, examination of partnerships was limited to the naming of allied university actors [83, 84]. For the first time, in-depth information about structural characteristics of a network promoting students’ health at university obtained from social network analysis was presented by Bachert et al. [85]. By analyzing 33 university actors and hundreds of ties in a network-analytical approach, key stakeholders were identified, network measures explored, and starting points for network development designated. Still, research into health-promoting universities is lacking, since no corresponding network has been examined so far for the processes underlying tie formation, network emergence, and maintenance.

### Objective and hypotheses

Therefore, the purpose of this study is to obtain insight into the mechanisms of cooperation between university actors in a health-promoting network and to identify the structural and attributive factors associated with establishing cooperation between actors in the observed network. This leads to the following research questions:

Which influence do individual attributes of the actors have on the probability of connections in a health-promoting university network?

What influence do structural properties of the network have on the probability of connections in a health-promoting university network?

Having answered these questions, a better understanding of how to build and develop successful partnerships in the future will be obtained.

**Fig. 1:**
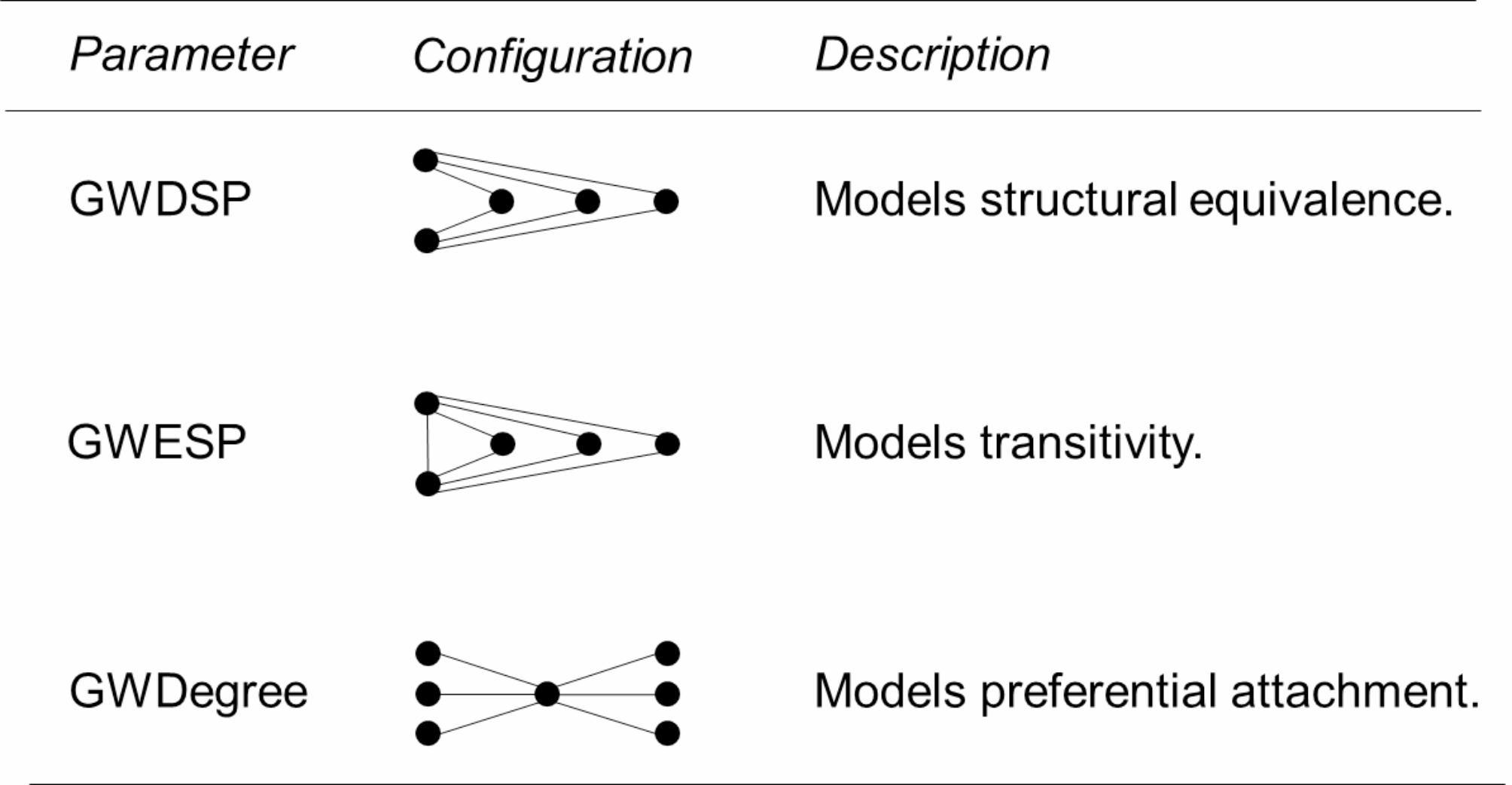
Description of included parameters

On this basis, we tested whether exogeneous (attributive) effects and specific endogenous (microstructure) configurations occur more often within an observed network. While the structural effects represent self-organizing characteristics of the network (see Fig. 1), the actor attribute effects refer to characteristics of the actors.

Considering the fact that organizational characteristics (e.g. perception of the importance of being part of a network) can cause higher activity in creating cooperative relationships to others [86], it was hypothesized that there are significant activity effects in health-promoting networks at university. Perception of the importance of being part of a network is a relational determinant of tie formation because it influences how actors perceive the benefits and costs of forming and maintaining ties within a network [87, 88]. These perceptions may have a significant impact on the dynamics of cooperation within the network.

- H1: Actors of health-promoting universities deemed competent regarding student health issues show a higher activity in forming cooperative ties.
- H2: Actors of health-promoting universities that consider student health issues to be important in general show a higher activity in forming cooperative ties.
- H3: Actors of health-promoting universities that are considered important regarding student health issues show a higher activity in forming cooperative ties.

Based on the principle of homophily, which states that interaction between similar actors occurs more frequently than between dissimilar actors [87], it was hypothesized that there are significant homophily effects in health-promoting networks at university. Homophily operates on the principles of attraction, familiarity, and shared interests, leading to the formation of cohesive social groups based on common traits [83]. It has been shown that the participation of students themselves (e.g. student groups or representative boards) is a crucial element in health-promoting networks at university [89].

- H4a: Student actors of health-promoting universities form more cooperative ties among each other.
- H4b: University units of health-promoting universities form more cooperative ties among each other.

In social network analysis, two nodes are considered structurally equivalent, if they are connected to the same actors in the network [90]. Due to the frequent occurrence of this process of network self-organization in different contexts, it was of interest whether this effect could also be observed for the available network data. Accordingly, it was tested whether there are significant structural equivalence effects (GWDSP – geometrically weighted dyad-wise shared partner, clustering) in health-promoting networks at university.

- H5: Actors of health-promoting universities form multiple 2-paths in the network (see GWDSP in Fig. 1).

Transitivity, the tendency for two nodes that share a cooperative tie to form complete triangles with other nodes in the network [91], is another common network phenomenon [92]. It is likely to also appear in health-promoting networks at university, where there is a tendency of actors to work in small group-like clusters [41]. Therefore, it was hypothesized that there are significant transitivity effects (GWESP – geometrically weighted edgewise shared partner, clustering) in health-promoting networks at university.

- H6: Actors of health-promoting universities form triplets of cooperation in the network (see GWESP in Fig. 1).

Preferential attachment, or in other words the so-called mechanism of cumulative advantage, is a process often encountered in social networks [93, 94]. It can be assumed that the network at hand also shows this specific characteristic, because coordinating lead actors are common in the field of health services [95], especially in health-promoting universities [85]. Since GWDegree is a parameter that accounts for preferential avoidance [96], a negative parameter value suggests centralization, meaning that ties from low- to high-degree actors are more likely. Nodes with a higher degree commonly have a stronger ability to capture links added to the network [97]. Consequently, we formulated the hypothesis that there are significant preferential attachment effects (GWDegree – geometrically weighted degree, centralization) in health-promoting networks at university.

- H7: Actors of health-promoting universities form more cooperative ties to popular actors (see GWDegree in Fig. 1).

### Theoretical background and methodological approach

Network theory posits that actors do not work in isolation but are embedded in a system, which is why their relationships with each other are considered here [52]. Social network analysis is an effective method for dealing with relational data. The present network analysis is part of the research area of organizational network analysis [98]. Organizations can be conceptualized as a network where organizational members or units (e.g. composed of key representatives of these organizations) are nodes that interact with each other, and form relationships [99]. These networks among organizational actors are called intra-organizational networks, in contrast to inter-organizational networks, which emphasize networks between different organizations [53, 100]. Moreover, the present network analysis is a so-called network coordination model, in which a bond describes the type of relationship that exists between two individuals or entities [101]. In contrast to this, network flow models refer to the flow of resources, information, or influence between individuals or entities. With a view to the research tradition, the present work can be classified to belong to the area of ​​cooperation, where combinations of nodes act as a unit and bond-based explanations of achievement are obtained [101].

ERGM (exponential random graph modeling) represents a stochastic network modeling approach [102], which allows to predict the probability of a link between any two network nodes while accounting for the assumption that ties in a network are dependent on the presence or absence of other relationships [88]. To test the hypotheses listed above, ERGM was applied to identify attributes of actors, relationships, and structures associated with cooperative relationships. From a mathematical point of view, ERGMs are probability distributions modeling the probability that a relation between actors exists on the basis of a linear function of predictors [86]:

$$P\left(X\right)=\frac{1}{\kappa \left(\theta \right)}{exp}\left(\sum _{i}{\theta }_{i}{s}_{i}\left(X\right)\right)$$

ERGMs explain the global pattern of an observed network $\left(X\right)$ as a function of statistical parameters ${\theta }_{i}$ and local microconfigurations ${s}_{i}\left(X\right)$. In turn, the probability of the observed network $\left(X\right)$ is expressed as a function of the local microconfigurations ${s}_{i}\left(X\right)$. A normalizing constant $\kappa \left(\theta \right)$ is included in the model formulation so that the probability of the observed network ranges between 0 and 1. Similar to regression, the observed network $\left(X\right)$ represents the criteria, the local microconfigurations ${s}_{i}\left(X\right)$ represent the predictors, and the corresponding statistical parameters ${\theta }_{i}$ indicate the importance of the local microconfigurations ${s}_{i}\left(X\right)$ in determining the global pattern of the observed network $P\left(X\right)$. The local microconfigurations ${s}_{i}\left(X\right)$ can represent both endogenous and exogenous microstructures. The statistical parameters ${\theta }_{i}$ and simultaneous consideration of other effects in the model allow conclusions to be drawn as to whether the specific local microconfigurations ${s}_{i}\left(X\right)$ occur more or less frequently in the observed network $\left(X\right)$ than expected by chance. So, if the number of local microconfigurations ${s}_{i}\left(X\right)$ found in the observed network $\left(X\right)$ is higher than the number expected when ties are formed randomly, there is evidence of the significance of the local microconfigurations ${s}_{i}\left(X\right)$ in explaining the global configuration of the observed network $\left(X\right)$. A positive (negative) local microconfigurations estimate ${s}_{i}\left(X\right)$ suggests the presence of a higher (smaller) number of these configurations in the network than expected by chance, which provides (no) evidence of this particular mechanism being associated with such configurations [102].

## Methods

### Measures

For the survey, a questionnaire (see Additional file 1) was developed based on previous work on health-related networks [54, 86, 103, 104]. The quantitative relational construct measured among the university actors was cooperation, operationalized as the type of cooperation. For this question, a list of the 33 actors was provided. Respondents were asked how they would describe their relationship with each of the 33 actors. The cooperation response scale included no cooperation (0); information sharing only (1); informal cooperation (loose cooperation to reach common objectives) (2); formal cooperation (close cooperation in a team to reach common objectives) (3); partnership (close cooperation for a longer time period, e.g., in several projects) (4). Respondents were additionally asked for the relevance of the other actors regarding health topics and the importance of the other actors to student health proper (on a five-point Likert scale from 1 = unimportant to 5 = very important).

### Sampling and data collection

Data were collected at a German university with more than 20,000 students. To identify all actors in student health at the university at hand, a multifaceted snowball sampling process was initiated [54, 103, 105]. This resulted in a final sample of 33 actors, who focus on understanding or promoting the health of students at university or who are potentially able to influence student health. It is a sociocentric network – more information on the setting, the sampling process, and the sample is given in Bachert et al. [85]. Organizational network data were collected during the 2019/2020 winter semester by highly structured face-to-face interviews from trained research assistants using an interview guide in an interactive format with actor and health topic lists and response scale cards [85]. The main representative of each of the 33 actors (generally, the executive director or, in some cases, a staff member who was more knowledgeable of the issue) received a personalized interview request for this purpose, including a cover letter explaining the research study and a privacy statement [85]. In the end, 28 of 33 actors completed the survey, corresponding to a response rate of 85%. 7 of the 33 actors were representatives of the organized student body (e.g. General Student Committee or Student Parliament), while the remaining 26 actors were conventional university units. Three of the 33 actors (student groups, deaneries, and institutes) represented a collective of various actors and were therefore not interviewed. The General Student Committee and the Student Working Group for Culture and Communication were not available for an interview. In total, 35 persons were interviewed, since the Institute of Sports and Sports Science (three respondents), the Central Scientific Institution for Key Competencies (five respondents), and the Student Support Service (two respondents) in their roles as central stakeholders in the context of student health had more than one respondent. The actors are more or less relevant to promoting students’ health. Some of them actually promote health (e.g. University Sports Center or Student Support Service), others provide health-related information (e.g. Center for Information and Counseling) or qualification opportunities (Central Scientific Institution for Key Competencies), and others are indirectly involved in aspects of student health, for example through the education of lecturers (e.g. Human Resources Development and Vocational Training). Study approval and execution are described in Bachert et al. [85].

### Data processing

Survey data gathered through the questionnaire were entered in SPSS 25 Statistical Package by study ID for accuracy checking, cleaning, and initial data exploration on the basis of a codebook, before data from the network questions were exported to Microsoft Excel for the creation of adjacency matrices. For the logistic models we dichotomized the cooperation variable as 0 = unlinked and information exchange only and 1 = informal cooperation, formal cooperation, and partnership. Only informal cooperation, formal cooperation, and partnership were kept, as they reflect viable types of relationship between actors and tend to be more consistent. To reconcile divergent response pairs, two techniques were used in UCINET: Reconstruction (when only one actor in the dyad provided a valid response to a question and the response given by the other actor in the pair was used; [106]) and symmetrizing (maximization was used to resolve rating discordances between two actors in a dyad). When both actors in the dyad did not give a valid response, it was treated as a missing value – and therefore recoded to 0 –, which was the case for 20 (5 non-interviewed actors × 4) of 1,056 ties, corresponding to a missing rate of < 2%. If multiple respondents were interviewed from one unit or group, we used the responses given by the person highest in the hierarchy.

### Data analysis

For the descriptive procedures, data were analyzed in UCINET 6. For an analysis of structural cohesion at the network level, various measures of network cohesion were calculated [51, 98]: Average degree (average number of edges per node in the graph), centralization (extent to which the graph shows a centralized structure), density (number of existing ties divided by the number of possible ties), fragmentation (extent to which the network is broken into fragments of unconnected nodes, dyads, and cliques), average distance (average number of steps along the shortest paths (geodesics) for all possible pairs of network nodes), and diameter (largest geodesic distance in the network). The network map representing cooperation between actors was visualized using GEPHI 0.9.2. ERGM analyses were performed with R version 4.1.2 (The R Foundation for Statistical Computing, https://www.r-project.org) using the statnet package.

We estimated the parameters of the exponential random graph model using Markov chain Monte Carlo (MCMC) methods. Model fit was assessed based on a comparison of AIC and BIC scores throughout model development and goodness-of-fit statistics for common network distributions. Predictors were classified into two categories: Structural predictors capturing aspects of local network structures and processes and node attributes accounting for organizational characteristics of the individual network members. Three stages of model building were performed. Alpha was increased in each case until AIC und BIC had the lowest value.

First, a null model (model 0) was created as baseline. It is a single parameter model, being essentially the network density, without any predictor that assumes equal probability for all edges in the network [107].

In a second step, organizational characteristics (type, importance, assessment of significance, and competence) were added as node attributes for model 1 to capture their effects on the likelihood of cooperation between actors.

- *Type of actor* was a dichotomous variable that indicates whether an actor is a student actor or a university actor. Student actors were used as the reference category.
- *Attributed importance* was a continuous variable reflecting actors tending to be perceived by the network as rather important vs. actors tending to be perceived as rather unimportant to student health. For this purpose, the rounded mean value on a five-point likert scale was used.
- *Assessment of significance of the health topics* was treated as a continuous variable. For this purpose, the mean value of all items on the basis of a five-point likert scale was included.
- *Attributed competence* is another dichotomous variable, which stands for the actors’ competence perceived by the network. The decision as to whether someone was competent was made according to whether the actor was deemed to be the competent in one of the thirteen health topics. Incompetent actors were used as the reference category.

Finally, model 2 was developed. In addition to nodes’ attributes and communication linkage, it also took structural patterns (preferential attachment, brokerage, and transitivity) into account when explaining the cooperation behavior between actors in order to uncover important aspects of network configuration. We included three commonly used geometrically weighted structural terms in model 2: GWDSP, GWESP, and GWDegree [108, 109].

The stepwise approach from model 0 to model 1 to model 2 has the advantage of gradually increasing the complexity of the model, improving the fit to the data, and facilitating the interpretation of the effects [110].

## Results

### Descriptive analysis

The analyzed network consists of 33 actors (see Additional file 2).

**Fig. 2:**
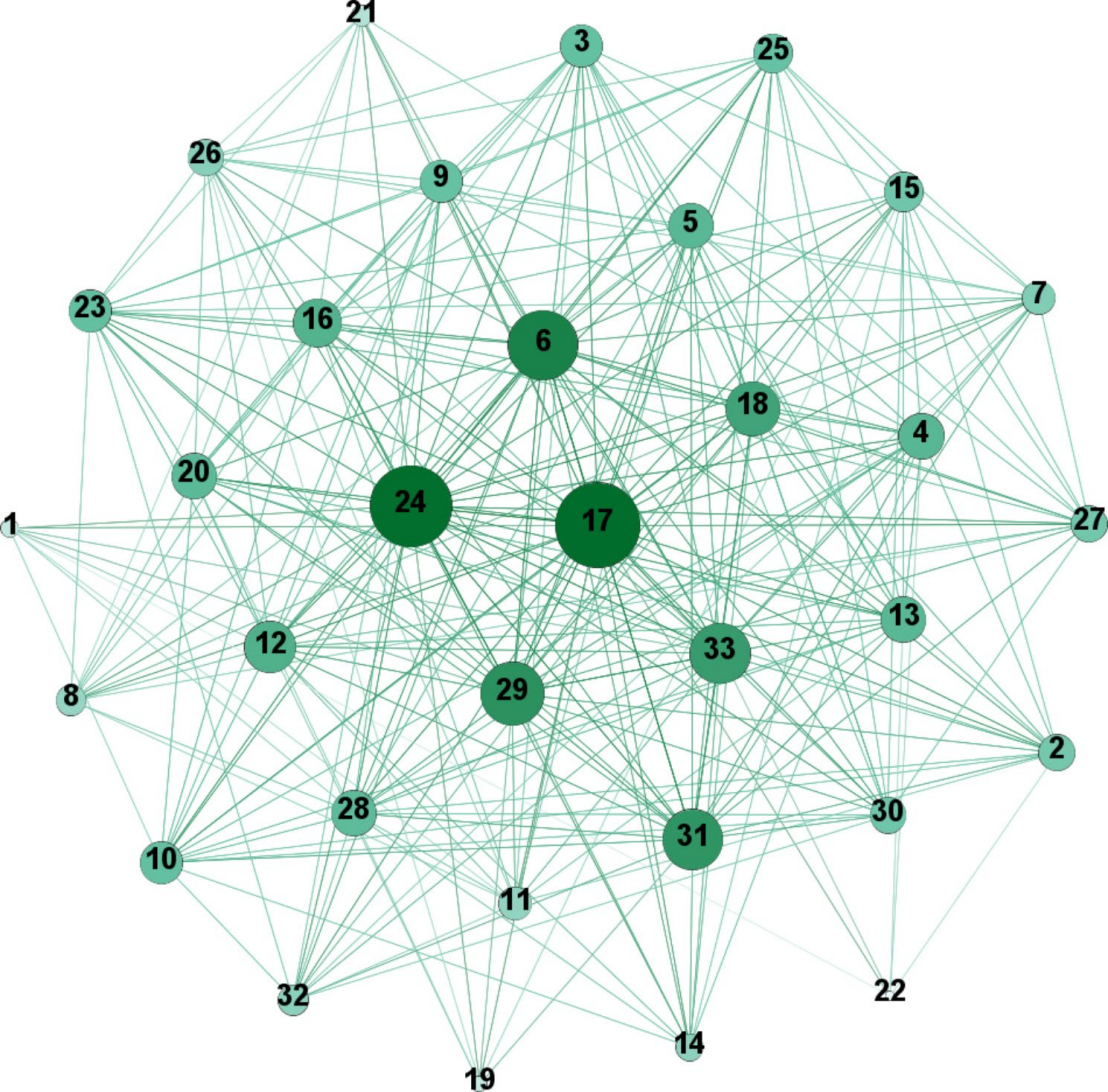
Cooperation network (node size represents degree centrality; node color represents eigenvector centrality)

566 of 1,056 possible ties of the network are realized, resulting in a relatively high density of 0.54. The network shows a flat, non-hierarchical structure, which is typical of the university context [111]. This structure is reflected by a low centralization (0.46) and a short average distance (1.46) with a low standard deviation (0.50), indicating that every actor can be reached by every other actor via one to two nodes. The largest geodesic distance in the network, which is expressed by diameter (2), is small. With regard to fragmentation, the network shows the non-existence of subgroups. The average degree is 17.2 (SD = 5.5), which means that every node is connected with more than half of the networks’ nodes on the average. Network measures for the cooperation network are reported in Table 1 and the network is visualized in Fig. 2.

**Table 1:** Network measures of the cooperation network

Measures	Cooperation network
Number of nodes	33
Number of ties	566
Average degree	17.15 (SD = 5.5)
Degree centralization	0.46
Density	0.54
Fragmentation	0
Average distance	1.46
Standard deviation distance	0.50
Diameter	2

### Exponential random graph models

**Fig. 3:**
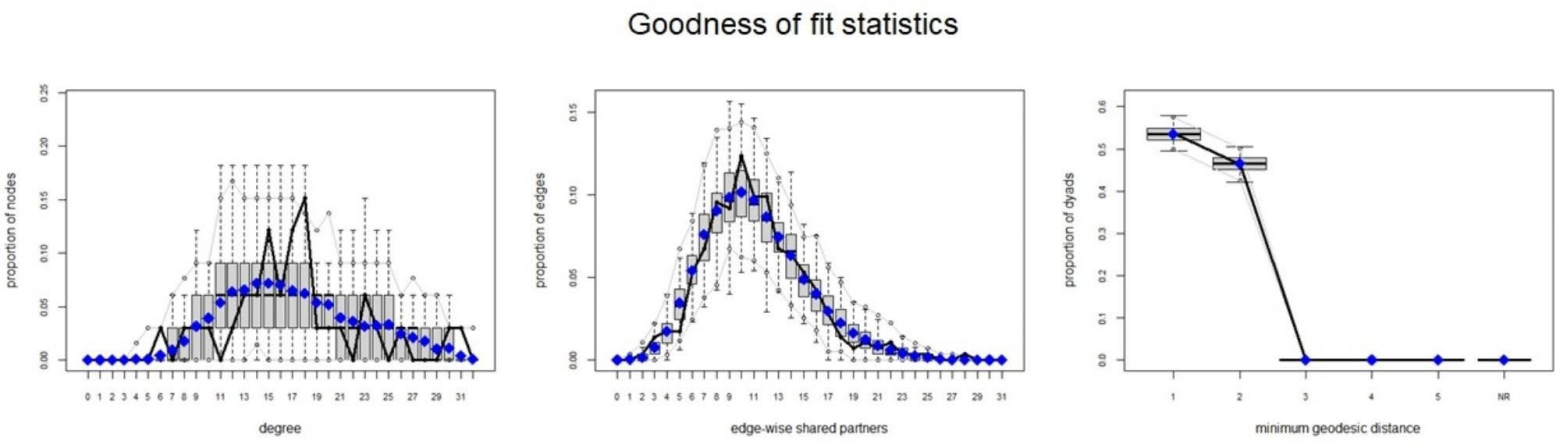
Goodness of fit statistics: The edge parameter, which describes the probability of a relationship taking into account the attributive and structural effects, is negative (-13.33; p < 0.05), since the existence of a relationship between two random actors is less likely than the absence of this relationship, suggesting that cooperation realized in the network is less than expected by chance

Goodness-of-fit statistics, where the observed network was compared to numerous networks simulated by the model, showed a sound model fit (see Fig. 3). The results of the estimated ERGM models are reported in Table 2. Only model 2 is described in more detail below, as is usual in ERGM research.

The significant positive estimate for attributed competence (0.32; p < 0.05) provides evidence of an activity effect – implying a higher activity in forming cooperative ties for competent actors. Hence, hypothesis 1 is confirmed.

The non-significant positive estimate for assessment of significance of the health topics (0.11; p > 0.05) provides no evidence of an activity effect – implying that actors, who consider student health issues to be more important in general, show no higher activity in forming cooperative ties. Hypothesis 2, hence, is not confirmed.

The non-significant negative estimate for attributed importance (-0.002; p > 0.05) provides no evidence of an activity effect – implying that actorsconsidered important with regard to student health show no higher activity in forming cooperative ties. Hypothesis 3 is not supported.

The significant positive estimates for the type of actor provide evidence of a homophily effect among student actors (1.31; p < 0.05) and among university actors (0.59; p < 0.05), indicating that being of the same type appears to be a predictor for cooperation in the analyzed network. Hypotheses 4a and 4b are confirmed. Due to model divergence, the activity effect is excluded for the type of actor.

The significant positive estimate for GWDSP (0.22; p < 0.05) provides evidence of a structural equivalence effect – implying a tendency for clustering, that is, members of dyads in the network tend to share ties with the same sets of partners. Hypothesis 5 is confirmed.

The significant positive estimate for GWESP (0.24; p < 0.05) provides evidence of a transitivity effect – implying a tendency for path closure among the actors, which means that network members tend to form complete triangles with other network members. Hypothesis 6 is confirmed.

The significant positive estimate for GWDegree (5.40; p < 0.05) provides evidence of a preferential avoidance effect rather than a preferential attachment effect – implying a tendency for a more even share of degree among actors. Hypothesis 7 is not supported.

**Table 2:** Stochastic models predicting the probability of cooperation between two actors in the network (* = p < 0.05)

	Model 0: Null model		Model 1: Attributive predictors		Model 2: Attributive and structural predictors	
Coefficient	Estimate	Std. Error	Estimate	Std. Error	Estimate	Std. Error
Cooperation (edges)	0.14	0.09	-2.81	1.55	-13.33*	3.86
***Attributive predictors***						
Student type of actor (homophily)			1.19*	0.48	1.31*	0.44
University unit type of actor (homophily)			0.83*	0.21	0.59*	0.18
Attributed importance (activity)			-0.02	0.14	-0.002	0.08
Assessment of significance of the health topics (activity)			0.26	0.14	0.11	0.10
Attributed competence (activity)			0.98*	0.18	0.32*	0.14
***Structural predictors***						
GWDSP (structural equivalence)					0.22*	0.07
GWESP (transitivity)					0.24*	0.07
GWDegree (centralization)					5.40*	2.48
***Model fit***						
AIC	731		667		732	
BIC	736		693		622	

## Discussion

### Summary of findings and interpretation

The aims of this study were to describe a university network for health promotion, to assess the likelihood of cooperation between the network’s members, and to identify the factors associated with cooperation. We analyzed data collected from 33 actors of a German university, who had established 566 relationships among each other. The network is a high-density, decentralized network.

As regards the first research question, it can be seen that attributed competence predicts cooperation. Unexpectedly, attributed importance and assessment of significance of the health topics do not seem to be significantly associated with establishing cooperation. But homophily effects are present among student actors and among university actors. Since student actors tend to cooperate with other student actors and university actors tend to cooperate with like actors, it is important to initiate more cooperative endeavors between these two types of actors.

As regards the second research question, the structural predictors GWDSP, GWESP, and GWDegree are positive and statistically significant. Hence, they are essential in determining the likelihood of cooperation between actors involved in the network. The GWDegree effect can be attributed primarily to the interconnectedness of the vast majority of actors. The positive significant GWESP effect indicates that cooperation partly takes place in small triangular and trustworthy clusters that may also bear responsibility for the health of students.

Health promotion focuses on shaping the social preconditions of health. To a large extent, these conditions are created by organizations for a lot of people, which is why targeted setting-based interventions are an important strategy. As regards university students, this approach is complicated because their fluid membership status results in an unclear legal basis. Young people take on different roles at university. They can be students as course participants and examinees, employees as student and research assistants, and customers when using certain university services. On the other hand, fluctuation among students is relatively high. Universities therefore face the challenge of sustainably promoting health and personal development within a relatively short period of time. In order to promote students’ health more effectively, the number of network members, the number of relationships, and the intensity of existing relationships could be increased [49]. According to the theoretical concept of *strength of weak ties*, strengthening of existing weak ties leads to possible higher levels of diversity in the network. According to the theoretical concept of *structural holes*, by contrast, closing of gaps between actors having complementary sources of information reduces redundancy by adding isolates to new other subgroups. However, these efforts can also lead to the following challenges [112]: Increased interorganizational competition, time and resource investment with little benefit to members, worsening benefit-cost-ratio or reduced efficiency after reaching a certain network size, network opposition and professional protectionism, ambiguity or uncertainty relating to accountability mechanisms, and coercion or manipulation of weaker network members by more powerful ones.

A certain form of network governance, in other words a conscious decision for the creation of an organizational structure to coordinate all actions relating to the aims of a network, is required to utilize the benefits of cooperation among network members [113, 114]. The network at hand shows characteristics of a “participant-governed” network. Such a network is governed by virtually all units coordinating activities and making decisions (although a handful of actors play a special role in it as a kind of “leading group”). Such networks are common in the field of health services to build community capacity [95]. However, thought could still be given to whether a change in the governance approach might be useful. In “lead organization-governed” networks, for example, the network is led and coordinated by a legitimized central actor trusted by others [113]. This form of governance can certainly also be encountered in health-promoting universities and is considered advantageous by some experts [48]. It also works with low commitment levels of the network members and is best suited for a moderate number of involved actors. To increase the efficiency of the network, a “network administrative organization” may be considered, where governance is carried out externally by an independent unit specifically established to govern the network only [113]. This approach best fits networks with moderate density and centralization, moderate to many network participants, and a moderately high goal consensus. It should be noted that such an alliance of different actors, most of whom are not professionally involved in practical health promotion, may not be motivated to commit to promoting students’ health in the long term. In addition, certain knowledge and skills need to be developed and proper moderation by intermediary units with spatial equipment, material and personnel resources is often required for effective health promotion.

Network development efforts can be connected to other highly regarded approaches in the field of health promotion, such as *community-based participatory research for health* [115], in which equal cooperation between professionals and recipients is a priority. *Capacity building*, which postulates building infrastructure and collaborative partnerships for health promotion in organizations [116] or the idea of *integrating health promotion services* to disseminate them more effectively through the network [117] are two more examples in this context. Anyway, change in an organization can only be embraced if actors can simultaneously rely on continuity [118]. The focus of network development must therefore be on both: On what needs to be preserved (maintaining and enabling networks) and on what needs to be changed (further developing and stimulating new networks). Another crucial point is connecting health promotion to the original objectives of the organization and its actors (*health co-benefits*) to incorporate health as a goal and anchoring it. Referring to the university this means relating health promotion to teaching and research in order to support and maintain health and promote the well-being of university students.

There are a number of ways to identify key actors and potential for development in a network, such as focus groups or knowledge mapping [119]. Actor identifications generally pursue the questions of who is or should be involved, who is related to whom, and who is influential. ERGM can also answer the question of how the relationships are established in the first place. Moreover, the method of social network analysis can be used as both an analysis and intervention tool. Face-to-face interviews in particular are ideal for collecting network data while informing, raising awareness, and encouraging networking. However, social network analysis also involves numerous pitfalls, some of which are outlined in the limitations section below.

### Limitations and future direction

This study is the first to quantitatively examine with the help of ERGM a network for health promotion in a university setting. Nonetheless, it is a snapshot at one timepoint and comes with several limitations: The network boundary drawing and the chosen sampling process may bias the actors interviewed. The survey questions and response items may have limitations, as they have not been tested for validity and reliability. A bias in reporting data is another possible limitation in this network analysis, since it is based on a single individual’s interpretation of the interconnectedness of an organizational unit. Furthermore, the response behavior may be characterized by social desirability. Future studies should avoid the limitations mentioned above, periodically track the network’s evolution of cooperation to move closer to causal inference, and particularly examine barriers and facilitators of cooperation.

## Conclusion and transferability

This network analysis is the first to quantitatively examine a network for health promotion in a university setting with the help of exponential random graph models. For the first time, the results of this study provide an understanding of how a network promoting health at university is structured and which mechanisms of cooperation are at work. However, the results cannot simply be transferred to other universities, but they can definitely be used to further develop the network at hand and provide starting points for sustained network development at other universities. At the time of the survey, the university health promotion efforts for students mainly focused on the areas of exercise (e.g. sports courses), mental health (e.g. psychosocial counseling), and offerings for vulnerable student groups (e.g. counseling for students with disabilities). In addition, a health promotion project sponsored by a German insurance company was initiated just before the network analysis to implement a health management system for students, the primary goal of which was to coordinate the existing offers and develop new ones. Student groups (e.g. General Student Committee) were already offering independent counseling services for vulnerable student groups (e.g. disadvantage compensation counseling for students with children) prior to that project and were included as full members of the project’s steering and working group in order to ensure their participation in the health promotion process. Knowing the factors that influence the network structure, here the conditions of cooperation, results in opportunities to encourage empowerment among the actors. The results show the significance of members of the university`s executive board and health-related disciplines (e.g. Institute of Sports and Sports Science) as key stakeholders and the relevance of crosswise integration of health promotion through major business units of the university (e.g. Central Scientific Institution for Key Competencies). These actors can play a crucial role in disseminating information, promoting healthy behaviors, and encouraging the adoption of preventive measures to enhance students’ health. The network analysis highlights potential collaboration opportunities between different actors. Efforts within a health-promoting university network may be facilitated by increasing the number of network members or the number of relationships and intensifying existing relationships [120]. In the present network, it can be seen that student groups are comparatively underrepresented with regard to cooperation, which is why their participation should be strengthened in order to utilize the potential of these subordinate actors. The results can also be used as a starting point to make an informed decision on the governance of the health-promoting network and increase its effectiveness [113]. In the future, social network analysis may also take a place as a new form of structural evaluation in health promotion that, compared to traditional evaluation approaches, focuses on documenting structural changes rather than on simply counting program activities or mapping processes [120].

### Electronic supplementary material

Below is the link to the electronic supplementary material.

Supplementary Material 1

Supplementary Material 2

## Data Availability

The datasets generated for this study are available on request to the corresponding author.

## References

[1] Stewart-Brown S, Evans J, Patterson J, Petersen S, Doll H, Balding J, Regis D (2000). The health of students in institutes of higher education: an important and neglected public health problem?. J Public Health Med.

[2] Dooris M, Doherty S (2010). Healthy universities: current activity and future directions–findings and reflections from a national-level qualitative research study. Glob Health Promot.

[3] Aceijas C, Waldhäusl S, Lambert N, Cassar S, Bello-Corassa R (2017). Determinants of health-related lifestyles among university students. Perspect Public Health.

[4] Haas J, Baber M, Byrom N, Meade L, Nouri-Aria K (2018). Changes in student physical health behaviour: an opportunity to turn the concept of a healthy University into a reality. Perspect Public Health.

[5] Hurst CS, Baranik LE, Daniel F (2013). College student stressors: a review of the qualitative research. Stress Health.

[6] Ribeiro ÍJS, Pereira R, Freire IV, de Oliveira BG, Casotti CA, Boery EN (2018). Stress and quality of life among University students: a systematic literature review. Health Professions Education.

[7] Kaggwa MM, Kajjimu J, Sserunkuma J, Najjuka SM, Atim LM, Olum R (2021). Prevalence of burnout among university students in low- and middle-income countries: a systematic review and meta-analysis. PLoS ONE.

[8] Ibrahim AK, Kelly SJ, Adams CE, Glazebrook C (2013). A systematic review of studies of depression prevalence in university students. J Psychiatr Res.

[9] Vadeboncoeur C, Townsend N, Foster C (2015). A meta-analysis of weight gain in first year university students: is freshman 15 a myth?. BMC Obes.

[10] Peltzer K, Pengpid S, Samuels TA, Özcan NK, Mantilla C, Rahamefy OH (2014). Prevalence of overweight/obesity and its associated factors among university students from 22 countries. Int J Environ Res Public Health.

[11] Imdad F (2016). Prevalence of low back Pain among the undergraduate students of Isra University Karachi campus. Int J Physiotherapy.

[12] Anggiat L, Hon WHC, Baait SN. The incidence of low back pain among university students. Pro-Life. 2018;5.

[13] Jiang X-l, Zheng X-y, Yang J, Ye C-p, Chen Y-y (2015). Zhang Z-g, Xiao Z-j. A systematic review of studies on the prevalence of insomnia in university students. Public Health.

[14] Jahrami H, Dewald-Kaufmann J, Faris M’eA-I, AlAnsari AMS, Taha M, AlAnsari N (2020). Prevalence of sleep problems among medical students: a systematic review and meta-analysis. J Public Health (Berl).

[15] Li L, Wang Y-Y, Wang S-B, Zhang L, Li L, Xu D-D (2018). Prevalence of sleep disturbances in chinese university students: a comprehensive meta-analysis. J Sleep Res.

[16] Wang X, Zhou HB, Sun JM, Xing YH, Zhu YL, Zhao YS (2016). The prevalence of migraine in university students: a systematic review and meta-analysis. Eur J Neurol.

[17] Wicki M, Kuntsche E, Gmel G (2010). Drinking at european universities? A review of students’ alcohol use. Addict Behav.

[18] Davoren MP, Demant J, Shiely F, Perry IJ (2016). Alcohol consumption among university students in Ireland and the United Kingdom from 2002 to 2014: a systematic review. BMC Public Health.

[19] Karam E, Kypri K, Salamoun M (2007). Alcohol use among college students: an international perspective. Curr Opin Psychiatry.

[20] Bernardo GL, Jomori MM, Fernandes AC (2017). Proenca, Rossana Pacheco da Costa. Food intake of university students. Rev Nutr.

[21] Irwin JD (2004). Prevalence of university students’ sufficient physical activity: a systematic review. Percept Mot Skills.

[22] Pengpid S, Peltzer K, Kassean HK, Tsala Tsala JP, Sychareun V, Müller-Riemenschneider F (2015). Physical inactivity and associated factors among university students in 23 low-, middle- and high-income countries. Int J Public Health.

[23] Castro O, Bennie J, Vergeer I, Bosselut G, Biddle SJH (2020). How sedentary are University students? A systematic review and Meta-analysis. Prev Sci.

[24] Patterson F, Lerman C, Kaufmann VG, Neuner GA, Audrain-McGovern J (2004). Cigarette smoking practices among american college students: review and future directions. J Am Coll Health.

[25] Guerra FMRM, Costa CKF, Bertolini SMMG, Marcon SS, Parré JL (2017). Consumo de tabaco entre universitários: uma revisão sistemática Tobacco consumption among college students: a systematic review. R pesq cuid Fundam Online.

[26] Skidmore CR, Kaufman EA, Crowell SE (2016). Substance use among College Students. Child Adolesc Psychiatr Clin N Am.

[27] Papazisis G, Siafis S, Tsakiridis I, Koulas I, Dagklis T, Kouvelas D (2018). Prevalence of Cannabis Use among Medical students: a systematic review and Meta-analysis. SubstAbuse.

[28] Shao Y-j, Zheng T, Wang Y-q, Liu L, Chen Y, Yao Y-s (2018). Internet addiction detection rate among college students in the people’s Republic of China: a meta-analysis. Child Adolesc Psychiatry Ment Health.

[29] Mortier P, Cuijpers P, Kiekens G, Auerbach RP, Demyttenaere K, Green JG (2018). The prevalence of suicidal thoughts and behaviours among college students: a meta-analysis. Psychol Med.

[30] Kühn L, Bachert P, Hildebrand C, Kunkel J, Reitermayer J, Wäsche H, Woll A (2022). Health literacy among University students: a systematic review of cross-sectional studies. Front Public Health.

[31] Valenzuela RLG, Velasco RIB, Jorge MPPC (2023). Impact of COVID-19 pandemic on sleep of undergraduate students: a systematic literature review. Stress Health.

[32] López-Valenciano A, Suárez-Iglesias D, Sanchez-Lastra MA, Ayán C (2021). Impact of COVID-19 pandemic on University students’ physical activity levels: an early systematic review. Front Psychol.

[33] Vindegaard N, Benros ME (2020). COVID-19 pandemic and mental health consequences: systematic review of the current evidence. Brain Behav Immun.

[34] Dietz P, Reichel JL, Edelmann D, Werner AM, Tibubos AN, Schäfer M (2020). A systematic Umbrella Review on the epidemiology of Modifiable Health influencing factors and on Health promoting interventions among University students. Front Public Health.

[35] Fernandez A, Howse E, Rubio-Valera M, Thorncraft K, Noone J, Luu X (2016). Setting-based interventions to promote mental health at the university: a systematic review. Int J Public Health.

[36] Burke NJ, Joseph G, Pasick RJ, Barker JC (2009). Theorizing social context: rethinking behavioral theory. Health Educ Behav.

[37] Stokols D, Grzywacz JG, McMahan S, Phillips K (2003). Increasing the health promotive capacity of human environments. Am J Health Promot.

[38] Dooris M, Doherty S (2010). Healthy universities–time for action: a qualitative research study exploring the potential for a national programme. Health Promot Int.

[39] Newton J, Dooris M, Wills J (2016). Healthy universities: an example of a whole-system health-promoting setting. Glob Health Promot.

[40] Ewing B, Ryan M, Zarco EP (2007). A campus wellness program: accepting the challenge. J N Y State Nurses Assoc.

[41] Sarmiento JP (2017). Healthy universities: mapping health-promotion interventions. HE.

[42] Dooris M, Wills J, Newton J (2014). Theorizing healthy settings: a critical discussion with reference to healthy universities. Scand J Public Health.

[43] Dooris M (2001). The health promoting University: a critical exploration of theory and practice. HE.

[44] Dooris M (2006). Healthy settings: challenges to generating evidence of effectiveness. Health Promot Int.

[45] Batras D, Duff C, Smith BJ. Organizational change theory: implications for health promotion practice. Health Promot Int. 2014;dau098. 10.1093/heapro/dau098. 10.1093/heapro/dau098 25398838

[46] Poland B, Krupa G, McCall D (2009). Settings for Health Promotion: an Analytic Framework to guide intervention design and implementation. Health Promot Pract.

[47] Woulfe J, Oliver TR, Siemering KQ, Zahner SJ. Multisector partnerships in Population Health Improvement. Prev Chronic Dis. 2010;7. PMC2995601 20950526

[48] Seibold C, Steinke B, Nagel E, Loss J (2010). Erfolgsfaktoren auf dem Weg zur Gesunden Hochschule. Praev Gesundheitsf.

[49] Varda DM, Chandra A, Stern SA, Lurie N (2008). Core dimensions of connectivity in public health collaboratives. J Public Health Manag Pract.

[50] Dooris M, Powell S, Parkin D, Farrier A (2021). Health promoting universities: effective leadership for health, well-being and sustainability. HE.

[51] Poghosyan L, Lucero RJ, Knutson AR, Friedberg W, Poghosyan M (2016). Social networks in health care teams: evidence from the United States. J Health Organ Manag.

[52] Wasserman S, Faust K. Social Network Analysis. Cambridge University Press; 2012.

[53] Wäsche H, Dickson G, Woll A, Brandes U (2017). Social network analysis in sport research: an emerging paradigm. Eur J Sport Soc.

[54] Buchthal OV, Taniguchi N, Iskandar L, Maddock J (2013). Assessing state-level active living promotion using network analysis. J Phys Act Health.

[55] An R, Loehmer E, Khan N, Scott MK, Rindfleisch K, McCaffrey J (2017). Community partnerships in healthy eating and lifestyle promotion: a network analysis. Prev Med Rep.

[56] Schoen MW, Moreland-Russell S, Prewitt K, Carothers BJ (2014). Social network analysis of public health programs to measure partnership. Soc Sci Med.

[57] McKinney MM, Morrissey JP, Kaluzny AD (1993). Interorganizational exchanges as performance markers in a community cancer network. Health Serv Res.

[58] Ramanadhan S, Salhi C, Achille E, Baril N, D’Entremont K, Grullon M (2012). Addressing cancer disparities via community network mobilization and intersectoral partnerships: a social network analysis. PLoS ONE.

[59] Valente TW, Coronges KA, Stevens GD, Cousineau MR (2008). Collaboration and competition in a children’s health initiative coalition: a network analysis. Eval Program Plann.

[60] Mulroy EA (1997). Building a neighborhood network: interorganizational collaboration to prevent child abuse and neglect. Soc Work.

[61] Valente TW, Fujimoto K, Palmer P, Tanjasiri SP (2010). A network assessment of community-based participatory research: linking communities and universities to reduce cancer disparities. Am J Public Health.

[62] Weiner BJ, Alexander JA (1998). The Challenges of governing Public-Private Community Health partnerships. Health Care Manage Rev.

[63] Franco ZE, Ahmed SM, Maurana CA, DeFino MC, Brewer DD (2015). A Social Network Analysis of 140 community-academic partnerships for Health: examining the healthier Wisconsin Partnership Program. Clin Transl Sci.

[64] Lang JE, Anderson L, James L, Sharkey J, Belansky E, Bryant L et al. The Prevention Research Centers Healthy Aging Research Network. Prev Chronic Dis. 2005;3. PMC1500966 16356370

[65] Kaluzny AD, Zuckerman HS, Rabiner DJ (1998). Interorganizational factors affecting the delivery of primary care to older Americans. Health Serv Res.

[66] Bolland JM, Wilson JV (1994). Three faces of integrative coordination: a model of interorganizational relations in community-based health and human services. Health Serv Res.

[67] Kwait J, Valente TW, Celentano DD (2001). Interorganizational relationships among HIV/AIDS service organizations in Baltimore: a network analysis. J Urban Health.

[68] Harris JK, Jonson-Reid M, Carothers BJ, Fowler P (2017). The structure of Policy Networks for Injury and Violence Prevention in 15 US Cities. Public Health Rep.

[69] Provan KG, Milward HB (1995). A preliminary theory of Interorganizational Network Effectiveness: a comparative study of Four Community Mental Health Systems. Adm Sci Q.

[70] Becker T, Leese M, McCrone P, Clarkson P, Szmukler G, Thornicroft G (1998). Impact of community mental health services on users’ social networks. PRiSM psychosis study. 7. Br J Psychiatry.

[71] Nakao K, Milazzo-Sayre LJ, Rosenstein MJ, Manderscheid RW (1986). Referral patterns to and from inpatient psychiatric services: a social network approach. Am J Public Health.

[72] Tausig M (1987). Detecting cracks in mental health service systems: application of network analytic techniques. Am J Community Psychol.

[73] Timm I, Rapp S, Jeuter C, Bachert P, Reichert M, Woll A, Wäsche H (2021). Interorganizational networks in Physical Activity Promotion: a systematic review. Int J Environ Res Public Health.

[74] Wäsche H, Wolbring L, Woll A (2021). Physical activity promotion in an urban district: analyzing the mechanisms of interorganizational cooperation. PLoS ONE.

[75] Wolbring L, Schmidt SCE, Niessner C, Woll A, Wäsche H (2022). Community networks of sport and physical activity promotion: an analysis of structural properties and conditions of cooperation. BMC Public Health.

[76] Provan KG, Harvey J, de Zapien JG (2005). Network structure and attitudes toward collaboration in a community partnership for diabetes control on the US-Mexican border. J Health Organ Manag.

[77] Luke DA, Harris JK, Shelton S, Allen P, Carothers BJ, Mueller NB (2010). Systems analysis of collaboration in 5 national tobacco control networks. Am J Public Health.

[78] Harris JK, Luke DA, Burke RC, Mueller NB (2008). Seeing the forest and the trees: using network analysis to develop an organizational blueprint of state tobacco control systems. Soc Sci Med.

[79] Fujimoto K, Volente TW, Pentz MA, NETWORK STRUCTURAL INFLUENCES ON, THE ADOPTION OF EVIDENCE-BASED PREVENTION IN COMMUNITIES (2009). J Community Psychol.

[80] Krauss M, Mueller N, Luke D. Interorganizational Relationships within State Tobacco Control Networks: a Social Network Analysis. Prev Chronic Dis. 2004;1. PMC1277948 15670440

[81] Eisenberg M, Swanson N (1996). Organizational network analysis as a tool for program evaluation. Eval Health Prof.

[82] Phillips SD (1991). Meaning and structure in Social Movements: mapping the network of National Canadian Women’s Organizations. Can J Pol Sci.

[83] Ferreira FMPB, Brito IS, Santos MR (2018). Health promotion programs in higher education: integrative review of the literature. Rev Bras Enferm.

[84] Suárez-Reyes M, van den Broucke S (2016). Implementing the health promoting University approach in culturally different contexts: a systematic review. Glob Health Promot.

[85] Bachert P, Wäsche H, Albrecht F, Hildebrand C, Kunz AM, Woll A (2021). Promoting students’ health at University: key stakeholders, Cooperation, and Network Development. Front Public Health.

[86] Wäsche H (2015). Interorganizational cooperation in sport tourism: a social network analysis. Sport Manage Rev.

[87] McPherson M, Smith-Lovin L, Cook JM (2001). Birds of a feather: Homophily in Social Networks. Annu Rev Sociol.

[88] Harris J. An introduction to Exponential Random Graph modeling. 2455 Teller Road, Thousand Oaks California 91320 United States. SAGE Publications, Inc; 2014.

[89] Gürster A, Helten J, Tittlbach S (2021). Transdisziplinäre Forschung in der Gesundheitsförderung bei Studierenden – ein systematisches review. Präv Gesundheitsf.

[90] Borgatti SP, Grosser TJ. Structural Equivalence: Meaning and Measures. In: International Encyclopedia of the Social & Behavioral Sciences: Elsevier; 2015. p. 621–625. 10.1016/B978-0-08-097086-8.43120-X.

[91] Newman ME (2001). Clustering and preferential attachment in growing networks. Phys Rev E Stat Nonlin Soft Matter Phys.

[92] Robins G, Lewis JM, Wang P (2012). Statistical Network Analysis for analyzing policy networks. Policy Stud J.

[93] Schnegg M, Krempel L, Borgatti SP, Hennig M (2012). Studying social networks: a guide to empirical research.

[94] D’Souza RM, Borgs C, Chayes JT, Berger N, Kleinberg RD (2007). Emergence of tempered preferential attachment from optimization. Proc Natl Acad Sci U S A.

[95] Provan KG, Kenis P (2008). Modes of Network Governance: structure, management, and effectiveness. J Public Adm Res Theor.

[96] Hunter DR (2007). Curved Exponential Family Models for Social Networks. Social Networks.

[97] Robins G, Snijders T, Wang P, Handcock M, Pattison P (2007). Recent developments in exponential random graph (p*) models for social networks. Social Networks.

[98] Luke DA, Harris JK (2007). Network analysis in public health: history, methods, and applications. Annu Rev Public Health.

[99] Brass DJ, Galaskiewicz J, Greve HR, Tsai W (2004). Taking Stock of Networks and Organizations: a Multilevel Perspective. AMJ.

[100] Baum JAC (2017). The blackwell companion to Organizations.

[101] Borgatti SP, Halgin DS (2011). On Network Theory. Organ Sci.

[102] Robins G, Pattison P, Kalish Y, Lusher D (2007). An introduction to exponential random graph (p*) models for social networks. Social Networks.

[103] Brownson RC, Parra DC, Dauti M, Harris JK, Hallal PC, Hoehner C (2010). Assembling the puzzle for promoting physical activity in Brazil: a social network analysis. J Phys Act Health.

[104] Slonim AB, Callaghan C, Daily L, Leonard BA, Wheeler FC, Gollmar CW, Young WF. Recommendations for integration of Chronic Disease Programs: are your programs linked? Prev Chronic Dis. 2007;4. PMC1893132 17362625

[105] Guldbrandsson K, Nordvik MK, Bremberg S (2012). Identification of potential opinion leaders in child health promotion in Sweden using network analysis. BMC Res Notes.

[106] Huisman M. Imputation of Missing Network Data: some simple procedures. In: Alhajj R, Rokne J, editors. Encyclopedia of Social Network Analysis and Mining. New York, NY: Springer New York; 2014. pp. 707–15. 10.1007/978-1-4614-6170-8_394.

[107] Goodreau SM (2007). Advances in Exponential Random Graph (p*) Models Applied to a large Social Network. Social Networks.

[108] Hunter DR, Handcock MS, Butts CT, Goodreau SM, Morris M (2008). ergm: A Package to Fit, simulate and diagnose exponential-family models for networks. J Stat Softw.

[109] Lusher D, Koskinen J, Robins G (2012). Exponential Random Graph Models for Social Networks.

[110] Snijders TAB (2011). Statistical models for Social Networks. Annu Rev Sociol.

[111] Hüther O, Krücken G (2018). Higher education in Germany—Recent developments in an International Perspective.

[112] Glandon D, Paina L, Hoe C (2021). Reflections on benefits and challenges of longitudinal organisational network analysis as a tool for health systems research and practice. BMJ Glob Health.

[113] Wäsche H. Interorganisational network governance in sport. In: Winand M, Anagnostopoulos C, editors. Research handbook on sport governance. Cheltenham, UK, Northampton, MA, USA: Edward Elgar Publishing; 2019. pp. 202–15. 10.4337/9781786434821.00021.

[114] Provan KG, Fish A, Sydow J (2007). Interorganizational networks at the Network Level: a review of the empirical literature on whole networks. J Manag.

[115] Wallerstein N, Duran B, Oetzel JG, Minkler M (2018). Community-based participatory research for health: advancing social and health equity.

[116] Liberato SC, Brimblecombe J, Ritchie J, Ferguson M, Coveney J (2011). Measuring capacity building in communities: a review of the literature. BMC Public Health.

[117] Mays GP, Scutchfield FD, Bhandari MW, Smith SA (2010). Understanding the organization of public health delivery systems: an empirical typology. Milbank Q.

[118] Paton K, Sengupta S, Hassan L (2005). Settings, systems and organization development: the healthy living and Working Model. Health Promot Int.

[119] Reed MS, Graves A, Dandy N, Posthumus H, Hubacek K, Morris J (2009). Who’s in and why? A typology of stakeholder analysis methods for natural resource management. J Environ Manage.

[120] Valente TW, Palinkas LA, Czaja S, Chu K-H, Brown CH (2015). Social network analysis for program implementation. PLoS ONE.

