# Supplementary material for: Analyzing mechanisms of interdisciplinary cooperation in promoting students’ health at university
Source: BMC Public Health. 2023 Oct 3;23:1911. doi: 10.1186/s12889-023-16786-2 (PMC10548763; doi:10.1186/s12889-023-16786-2)
Supplement: Supplementary file 1 — Supplementary Material 1 [file 12889_2023_16786_MOESM1_ESM.pdf]

Additional file 1. Questionnaire

Question 1

Original:

Wie beurteilen Sie die Bedeutung nachfolgender Themenbereiche im Hinblick auf die Gesundheit der Studierenden?

Die Skala geht von 1=unbedeutend, 2=weniger bedeutend, 3=unentschieden, 4=bedeutend bis 5=sehr bedeutend.

Themenbereiche: Campusgestaltung; Curricula der Studiengänge; Ernährungsangebote oder -beratung; Gebäude-, Arbeitsplatzgestaltung und Raumausstattung; Leistungs- und Gesundheitsdiagnostik; Schlüsselqualifikation, Qualifizierungs- und Weiterbildungsangebote; Sozialberatung; Sport-, Bewegungs- oder Entspannungsangebote; Stressbewältigung; Studienberatung; Studienorganisation; Suchtmittelberatung; Verhaltenscodex auf dem Campus

English translation:

How would you rate the importance of the following topics with regard to student health?

The scale goes from 1=insignificant, 2=less significant, 3=undecided, 4=significant to 5=very significant.

Topics: Campus design; Curriculum; Nutrition; Workplace design; Health diagnostics; Key qualification and further education; Social counseling; Sports and relaxation; Stress management; Study counseling; Study organization; Addiction counseling; Campus safety

Question 2

Original:

Wie würden Sie Ihre Beziehung zu den nachfolgenden Akteuren beschreiben?

Beurteilen Sie den Grad Ihrer Zusammenarbeit bitte auf einer Skala von 1=es findet keine Zusammenarbeit statt, 2=es findet lediglich ein Austausch von Informationen statt, 3=es findet eine informelle Zusammenarbeit statt (lose Zusammenarbeit um gemeinsame Ziele zu erreichen), 4=es findet eine formelle Zusammenarbeit statt (enge Zusammenarbeit in einem Team um gemeinsame Ziele zu erreichen) bis 5=es besteht eine Partnerschaft (enge Zusammenarbeit über einen längeren Zeitraum, z.B. in verschiedenen Projekten).

Akteure: siehe Additional file 2

English translation:

How would you describe your relationship with the following actors?

Please rate the degree of your cooperation on a scale from 0=there is no cooperation, 1=there is information sharing only, 2=there is informal cooperation (loose cooperation to reach common goals), 3=there is formal cooperation (close cooperation in a team to reach common goals) to 4=there is a partnership (close cooperation for a longer time period, e.g. in several projects).

Actors: see Additional file 2

Question 3

Original:

Wen erachten Sie im Zusammenhang mit den nachfolgenden Themenbereichen als den kompetentesten Ansprechpartner?

Beurteilen Sie bitte, welche Akteure in den nachfolgenden Themenbereichen Ihrer Meinung die höchste Kompetenz (im Sinne von fachlicher Eignung und Zuständigkeit) besitzen.

Themenbereiche: siehe Question 1

English translation:

Who do you consider to be the most competent actor in connection with the following topics?

Please assess which actors you consider to have the highest level of competence (in the sense of professional aptitude and responsibility) in the following topics.

Topics: see Question 1

Question 4

Original:

Für wie wichtig erachten Sie die nachfolgenden Akteure im Hinblick auf die Gesundheit der Studierenden?

Beurteilen Sie die Bedeutung der Akteure für die Förderung der Gesundheit von Studierenden bitte auf einer Skala von 1=unwichtig, 2=weniger wichtig, 3=unentschieden, 4=wichtig bis 5=sehr wichtig.

Akteure: siehe Additional file 2

English translation:

How important do you consider the following actors with regard to student health?

Please rate the importance of the actors for the promotion of student health on a scale from 1=unimportant, 2=less important, 3=undecided, 4=important to 5=very important.

Actors: see Additional file 2
